# Continuation of Kangaroo Mother Care when transitioning from facility to community: maternal and familial perspectives from South India

**DOI:** 10.1186/s12913-025-13615-7

**Published:** 2025-11-05

**Authors:** Sathya Jeganathan, Ingrid Kvestad, Santhini Rajendran, Signe Hjelen Stige

**Affiliations:** 1 Department of Pediatrics, Chengalpattu Medical College, Chengalpattu, India; 2 Centre for International Health, Department of Global Public Health and Primary Care, University of Bergen, Bergen, Norway; 3 Innlandet Hospital Trust, Lillehammer, Norway; 4 Health and Family Welfare Training Centre, Gandhigram Institute of Rural Health and Family Welfare, Gandhigram, India; 5 Department of Clinical Psychology, University of Bergen, Bergen, Norway

**Keywords:** Kangaroo Mother Care, Low-birth-weight infants, Family support, Breastfeeding challenges, Community health workers, Gender-inclusive practices

## Abstract

**Background:**

Kangaroo Mother Care (KMC) is an effective intervention shown to significantly lower neonatal morbidity and mortality, especially among low-birth-weight (LBW) infants. Despite its success, many families struggle to implement KMC effectively post-discharge.

**Method:**

This qualitative study employed a hermeneutical-phenomenological approach to explore the experiences of mothers and families practicing KMC after discharge from the hospital in South India. In-depth, semi-structured interviews were conducted with eight mothers, fathers, and grandmothers, focusing on their experiences with community KMC (cKMC) and their challenges in maintaining cKMC at home. All the data were transcribed verbatim and reflexive thematic analysis was done to identify the enablers and barriers to practice cKMC.

**Results:**

Three main themes emerged from the analysis: Breast Feeding - Persevering Despite Initial Difficulties, Kangaroo Mother Care - Seeing benefits but struggling to practice, and Family Boon or Bane - Family as a crucial context for cKMC practice. While encountering challenges, participants expressed a strong commitment to breastfeeding, persevering to breastfeed by expressing breast milk, getting donor milk and using skin-to-skin contact to increase the milk flow. KMC was adopted positively and benefitted from support by the healthcare team and infrastructure during hospital stay, but continuation at home was difficult due to inadequate counselling, lack of community follow-up and challenging home environment. Family emerged both as a support system and a source of a tension to KMC practice. While fathers and grandmothers actively supported KMC in hospital settings, post-discharge traditional gender norms and domestic responsibilities hindered continuity at home.

**Conclusion:**

To promote sustained family-inclusive cKMC practices, there is a need for structured education to empower all caregivers, including grandmothers and fathers. Adoption of gender inclusive terminology such as “Kangaroo Family Care” can help to dismantle gender-oriented perceptions and encourage participation of all family members. Engaging grandparents as champions of KMC can promote intergenerational support for families and improve the outcome of LBW newborns. Community health teams should strengthen through tailored training on antenatal counselling and post-discharge support.

**Supplementary Information:**

The online version contains supplementary material available at 10.1186/s12913-025-13615-7.

## Introduction

Preterm birth (gestational age < 37 weeks) and low-birth weight (< 2500 g) are the most important causes of infant mortality and disability due to the risk of long term neurodevelopmental disorders [1]. Low- and middle-income countries experience the highest burden of preterm births and low birth weight (LBW) infants [2]. In 2020, 14.7% of all babies globally were born with LBW [3]. Over half of preterm births worldwide occurred in just eight countries, with India accounting for 3.02 million preterm births annually, constituting 20% of the global total [4]. Extensive research has established a strong association between preterm birth and developmental disorders, including cerebral palsy [5–7], intellectual disabilities [8], attention deficit hyperactivity disorders (ADHD) [9], and developmental delays [10]. These alarming statistics highlight the urgent need to address neonatal care to meet the Sustainable Development Goal (SDG 3.2), which aims to reduce neonatal mortality to 12 per 1,000 live births by 2030 [11].

Kangaroo Mother Care (KMC) is an evidence-based intervention that significantly reduces neonatal morbidity and mortality, particularly among LBW and preterm infants. KMC comprises of essential components such as continuous skin-to-skin contact, exclusive breastfeeding, early discharge, and sustained follow-up care [12]. The World Health Organisation`s (WHO) global position paper on Kangaroo mother care - A transformative innovation in health care, defines the practice as including the following key features: continuous and prolonged (8–24 h per day, for as many hours as possible) skin-to-skin contact initiated immediately after birth, exclusive breastfeeding or breastmilk feeding when KMC is initiated in healthcare facilities, timely discharge either from the NICU to a lower level of care within the facility or discharge to home with skin-to-skin contact and close monitoring [13]. Developed in 1978 in South America, KMC is an essential modality of treatment for LBW babies. These practices have demonstrated effectiveness in reducing the incidence of neonatal infections, hypothermia, pain responses, and overall mortality [14, 15]. In India, where neonatal care remains a challenge due to the high prevalence of LBW and preterm births, the implementation of KMC was integrated into hospital settings with relative success [16]. However, the transition from hospital-based KMC to community-based care could represent several challenges that impede its effectiveness and sustainability as shown in previous studies [17, 18]. This could be particularly relevant in South India, where social, cultural, and systemic factors play a significant role in determining health outcomes.

The India Newborn Action Plan (INAP) established ambitious targets for KMC coverage, aiming for 35% by 2017, 50% by 2020, 75% by 2025, and 90% by 2030. To achieve these targets, the Indian government has established 1054 Sick Newborn Care Units (SNCUs) nationwide, which provide specialized care for small and sick newborns (Unicef India-2024). Tamil Nadu, a southern state with comparatively robust health infrastructure in an Indian context, has ensured the availability of newborn care facilities in all districts [16, 19]. Specifically, Chengalpattu Medical College Hospital (CMCH) has implemented quality improvement interventions in its neonatal intensive care unit (NICU) to increase the duration of KMC to over 16 h per day before discharge [20]. Despite these efforts, little is known about the transition of KMC from hospital to home settings.

This qualitative study explores the factors that enable or hinder the practice of community-based KMC in South India. Specifically, it examines caregivers’ experiences with KMC at the place of birth, the challenges they face in maintaining KMC practices at home, and their successes or struggles in sustaining the key KMC components, particularly breastfeeding and skin-to-skin contact. By understanding these dynamics, this study seeks to inform interventions that can bridge the gap between the hospital and home care, ensuring that the benefits of KMC reach the most vulnerable populations. Effective community-based KMC has the potential to significantly reduce neonatal mortality and improve health outcomes for LBW and preterm infants, contributing to the broader goal of enhancing newborn survival in South India and beyond.

## Method

### Study context and background

This study was motivated by the experiences of the primary author, working in the Paediatric department Intensive Care Unit (ICU) and the District Early Intervention Centre (DEIC) at the tertiary care hospital in Chengalpattu district. It was observed that many LBW infants discharged from the Neonatal Intensive Care Unit (NICU) frequently got readmitted in critical conditions. There are multiple factors which contribute to readmission such as infections, difficulties in feeding, poor thermal regulation, and socio-economic barriers for seeking medical assistance. The World Health Organization has emphasized that KMC is a proven intervention, and that when continued post-discharge it will protect the infant from sepsis, hypothermia and feeding issues (WHO, 2015). Hence, cKMC at home is considered an important element of the continuum of care package for LBW infants in the community. This study was designed to explore the challenges from the caregiver’s perspective and gain a deeper understanding of the enablers and barriers to continue KMC after discharge from the government health facilities.

### Study setting

The study was conducted in the Kancheepuram and Chengalpattu districts of South India. Chengalpattu Medical College Hospital (CMCH) serves as a tertiary care centre, offering free medical services to residents of both districts, where approximately 60% of deliveries occur. High-risk mothers are routinely referred to CMCH, and both LBW and preterm infants born at CMCH or referred from outside are admitted to the NICU. As per the Indian Facility Based Newborn Care (FBNC) guidelines [21], all LBW infants are mandatory to have KMC practice in the hospital and the families are counselled to continue KMC at home after discharge. Further the healthcare workers in the community (Village health nurse (VHN) from Primary Health Centres (PHC) and Integrated Child Development Scheme (ICDS) workers) are responsible to visit families at home to monitor and support KMC.

At CMCH, the protocol mandates the initiation of KMC in the NICU, with continued KMC practice in the step-down wards. The medical team emphasizes KMC throughout the hospital stay, supported by visual displays and daily counselling sessions, including guidance at the time of discharge. Notably, CMCH features a father KMC ward, equipped with semi-reclining cots to facilitate KMC for fathers, an amenity available only in select centres across the state.

Infants are discharged from CMCH upon reaching a weight of 1.6 kg and demonstrating consistent weight gain over three consecutive days. In the community, neonatal health services follow an interdisciplinary model. Primary Health Care (PHC) staff and ICDS personnel conduct home follow-ups to monitor the growth and development of discharged infants. These healthcare providers are trained in KMC and are responsible for assisting and monitoring KMC practices at the community level.

### Study design

This study adopted a constructivist paradigm to explore the subjective experiences of participants with KMC, acknowledging that their perceptions are shaped by their social and cultural contexts. A hermeneutical-phenomenological approach was chosen because it is well-suited to exploring the lived experiences of mothers and families, aligning with the study’s aim to deeply understand their perceptions and experiences of KMC after hospital discharge [22].

In-depth, semi-structured interviews were conducted within participants’ homes to capture authentic family perspectives on practicing KMC at home. The home setting was chosen to promote openness, comfort, and flexibility, enabling participants to share their experiences freely. Mothers could pause or continue the interviews according to their needs and the comfort of the mother-baby dyad. Each participant was assigned a unique code, and interviews were audio-recorded to ensure accurate transcription and facilitate cross-checking.

### Recruitment and participants

Collaboration was established with the discharge nurse/data entry operator in the NICU and the VHN at the community level, ensuring that participants informed consent and their voluntary participation were secured before inclusion in the study. Mothers of LBW infants were eligible to participate. A total of eight mothers, whose infants had been treated in the NICU and discharged, volunteered to participate after the project was explained to them. Purposive convenience sampling was done to ensure that there is representation from all strata of communities within Chengalpattu district (rural, semi-urban, urban and tribal). Mothers who were residing locally after discharge from hospital were invited. Mothers from different geographical areas within the district agreed to participate, and were included in the study. This enabled the inclusion of the divergent perspective while it also ensured the feasibility of follow-up.

Mothers were all married, between 21-30years, and predominantly from Hindu households. The educational status ranged from secondary school to post graduate study and with household income between 16,000 and 50,000 INR. Father’s occupations included farmer, mechanic and government clerk. The infants were preterm or LBW (880gms – 1880gms) with average NICU stay ranging from 7 to 60 days and all had been exposed to KMC during hospital stay.

In some cases, fathers and grandmothers, who were caretakers or present during the interviews, also participated in the interviews, providing additional insights into the caregiving dynamics and family support systems.

### Data collection and preparation of data for analysis

The interview guide (see complementary material) was translated to the native language Tamil, by bilingual experts who are familiar with the cultural contexts. Then the tools were back-translated into English to ensure cultural appropriateness and semantic equivalence, as per the prescribed guidelines for translation of tools and adaptation [13, 23]. Any discrepancy identified were reviewed by the first and second author, discussed and resolved to ensure consistency and accuracy. The tools were pilot tested with a small group of mothers who were not included in the study to assess cultural appropriateness, clarity, relevance, and final data collection was done after minor revisions.

In this region, it is customary for women to stay at their mother’s house for their first delivery, typically spanning 3 to 6 months post-delivery. From the second child onwards, mothers usually reside with their husband’s family. The VHN was contacted to determine whether each mother was at her maternal or marital home. Before traveling to the participants’ homes, the interview team confirmed each mother’s willingness to participate in the study.

The primary author (SJ) a child specialist did the interview, and she was assisted by a field researcher (nurse) to create a safe and confidential environment where mothers felt comfortable sharing their childcare experiences. During data collection, special attention was paid to ensure participant comfort and ethical standards. All participants provided informed consent before each interview, and they were assured that their responses would be treated with confidentiality and respect.

The in-depth interviews with mothers and family members lasted between 60 and 90 min, with an average duration of 70 min. Various techniques such as open-ended questions, active listening, probing, and other facilitative methods were used. The semi-structured interview guide adapted from Lydon et al. [24] was reviewed after initial interviews and adjusted to explore emerging themes more deeply.

The interviews were designed to prioritize the mothers’ narratives and life experiences. They guided the participants through their journey of care before childbirth, the experience of preterm delivery, the family’s experience immediately after birth, the transition to the NICU, and the KMC provided within the healthcare facility. The second part of the interview focused on the discharge process, the care provided at home, and the support received from family and the community. These interviews aimed to provide researchers insights into the challenges of KMC and to explore the behaviours and strategies families adopted to care for their LBW newborns. For a full overview of the interview guide, see supplementary materials.

The interviews were conducted in Tamil, the participants’ native language, and were audio-recorded. Additionally, the primary author took visual documentation of the physical surroundings in the homes where children were cared for. This included detailed notes on the space available for KMC and other relevant environmental factors. Audio recordings and transcriptions were securely stored on encrypted devices, and identifying information was removed during transcription to maintain confidentiality. By this process, it was ensured that all the members of the research team were able to discuss and engage in the analysis of the collected data.

The in-depth interviews were transcribed verbatim to maintain accuracy. To ensure precision and fidelity, these transcriptions were cross-checked against the audio recordings by two researchers (the third author and a research nurse), safeguarding the accuracy and integrity of the collected data.

The primary author (SJ) who has proficiency in English and is also a native speaker of Tamil language was involved in the interview, transcription and analysis of the data. Hence only forward translation was done, not backtranslation.

To protect anonymity and confidentiality, participants’ identities were concealed throughout the transcription and analysis phases. Precautionary measures were taken to protect the participants’ privacy and the sensitive information shared. For effective data management and organization, NVivo 12, a qualitative research software, was employed.

### Data analysis

The methodological approach grounded in the phenomenological tradition [22] emphasizes a deep alignment with participants’ experiences. This commitment to understanding participants’ lived realities informed the entire analysis process. The analytical process was guided by reflexive thematic analysis [25, 26], and began with a thorough review of the individual interview transcripts by all authors to decide on the analytical focus to be applied in the in-depth analysis of the material. This initial phase was exploratory and focused on identifying topics within the data that were of significant interest and aligned with the study’s aim. The emphasis was on capturing the lived experiences of mothers and families shared during the interviews. Through this process, the team decided on the following analytical focus: How do mothers understand and practice the components of KMC?

We adopted the reflexive thematic analysis approach, emphasizing the researcher’s role in generating knowledge. The framework proposed by Braun and Clarke [25, 26] was utilized for its flexibility and systematic approach to identifying themes within the qualitative data. After the initial reading phase, the authors convened for a reflective discussion on the first day of analysis. These discussions incorporated elements of both analysis and reflection, drawing on the researchers’ diverse backgrounds in child health, clinical psychology, and sociology. This interdisciplinary approach enriched the analysis and provided multiple perspectives.

The primary author (SJ) then coded the full material, under close supervision of SHS. The analytical focus was consistently used as a guide to which parts of the data were relevant to code. The coding process proceeded through several iterative rounds of supervision and coding to ensure good coding praxis. The codes were further examined for consistency, similarity, repetitiveness and differences. Further meeting with SHS, SJ, and IK were arranged to review the coded material and related codes were aggregated together and further organized to nodal categories. The team started the process of abstraction and construction of themes. After collaborative discussions the themes were further refined.

In the subsequent phase, the team further dissected the data into three distinct themes, developing a nuanced understanding of the diverse experiences and challenges encountered. The research team collaborated to achieve a consensus on descriptions of the experiences presented in each interview. Each phase was documented to ensure a high level of transparency, allowing readers to critically evaluate the applicability and credibility of the findings.

### Researchers

The research team had multidisciplinary experience, consisting of clinical care in neonatal medicine, psychology and community education. The primary author, a PhD candidate and clinical specialist of Pediatric Medicine, Chengalpattu Medical college, India. The second author from Innlandet Hospital Trust in Norway, is a child and adolescent psychologist and a research professor specialised in child nutrition and developmental research. The third author is Health education extension officer, who brings extensive experience in community education and family counselling. The fourth author is a professor of clinical psychology from University of Bergen, with vast experience in clinical training and qualitative research. All authors have professional experience for more than two decades and the intensive care nurses have more than 10 years of clinical experience and counselling.

### Reflexivity

With extensive experience in neonatal care, the primary author (SJ) acknowledges that clinical perspectives might influence interpretations. The primary author, who worked in the hospital, visited the homes of families and played the dual role of caregiver in the hospital and researcher in the field. Although the families treated the researcher (SJ) with respect (addressed as “Madam”), the impression is that they were not hesitant to narrate the real issues. Reflexive journaling was employed to critically evaluate how the author’s professional background, assumptions, and positionality influenced data collection and analysis, ensuring participants’ voices remained central.

### Ethical considerations

The study adhered to ethical principles outlined in the Declaration of Helsinki [27]. The research was approved by the Norwegian Regional Committee for Medical and Health Research Ethics (32413) and the Institutional Ethical Committee of Chengalpattu Medical College (ECR/774/INST/TN).

All participants were fully informed about the project and provided their signed informed consent before participating. Ethical handling of participants’ interviews and data was prioritized, ensuring confidentiality, respect, and integrity throughout the research process.

## Findings

The analysis resulted in the construction of the following themes, detailing mothers’ experiences with breastfeeding and the practice of skin-to-skin contact component of KMC.

1. *Theme 1: Breast Feeding – Persevering Despite Initial Difficulties*.
  Despite encountering various physical and emotional hurdles, many mothers demonstrated remarkable perseverance, motivated by the health benefits for their newborns
2. *Theme 2: Kangaroo Mother Care (KMC) – Seeing benefits but struggling to practice*.
  The barriers they face in consistent practice, such as physical discomfort, time constraints, cultural influence, lack of family and community support, were examined.
3. *Theme 3: Family Boon or Bane - Family as a crucial context for Community Kangaroo Mother Care (KMC) practice*.
  Examined the dual role of family as either facilitators or obstacles in the successful implementation of KMC.

### Theme 1: Breast feeding – persevering despite initial difficulties

Breastfeeding is one of the main three core components of KMC. The family members perspectives on initiating and sustaining it were examined as a part of exploring the continuum of KMC practice at home. All of the mothers intended to breastfeed for long term, also reported facing several obstacles to breastfeeding during the early postpartum period when many were separated from their baby at the hospital.

Breastfeeding was seen as health promoting for their babies for most participants, with bottle feeds being associated with health risks, based on advice by health professionals:

At the time of discharge doctor advised me, ‘Babies health is on your hand, you should continue KMC at home with breast feeding and the breast milk is the only nutrition that gives the energy to the baby.’ So, doctor informed me to continue breast milk for six months. (Participant 1)

However, most participants faced difficulties initiating breastfeeding during initial stages in NICU. Mothers were upset to see the tiny babies surrounded by lifesaving equipment but tried their best to give breast milk. The options available to the mothers were to strive hard for breast milk or switch to bottle feeds. Many participants were unable to give direct breastfeeding initially, since the baby was taken to intensive care:

Baby was there inside, and I was in the caesarean ward room. I was not giving milk for the first 10 days. (Participant 2)

In addition to the initial separation, many participants experienced difficulties due to inadequacy of milk, premature status of the infant resulting in weak sucking, physical pain and other limitations following delivery:

I had stitches on my body. --not able to sit. It was very painful. (Participant 1)

Mothers adopted various strategies to tackle milk inadequacy, namely borrowing from others, getting milk from the milk bank, but milk bank was not accessible for all. Mothers expressed, that they practiced skin to skin contact as a strategy to increase milk secretion.

Breast milk secreted well, while on KMC, it was good (participant 6)

Participants also expressed an understanding of how the milk secretion is depending on mother’s emotions and food intake. When the milk was perceived as inadequate, the families took special efforts to prepare foods believed to be galactagogues such as special fish (paal Sura/ Sura putu), garlic, homemade nutritious mix etc.:

In my family, they ask me to eat the special fish and I was adding the fish in the hospital. Special sea food called ‘paalsura’. It is available in special fish market. (Participant 8)

In contrast to the common belief that good food assists adequate milk secretion for the baby, a few mothers expressed concern about adverse consequence of more food, paving way for more milk which will choke the baby:

Eat more food, then there’ll be more milk. And when more milk comes through my breast, it will choke the baby’s breath. (Participant 2)

During the hospital stay as the babies’ condition improved, they were shifted from ICU to stepdown wards, families tried their best to adopt feeding practices from expressed breast milk through tube to direct breastfeeding:

The baby was always tube-feed, finally I came to the room with a screen, the tube was removed and paladai feed (cup feed) was given. The baby got the food through tube, through paladai (cup feed) and also tried breast milk. (Participant 6)

Most of the first-time mothers went to their parent’s home in accordance with the local cultural practice. Some mother felt milk secretion increased after arriving home. On the contrary a few felt the milk secretion decreased after going home – overwhelmed by the household responsibilities.

Mothers felt that it is important to breastfeed, and they persevered despite the challenges. Not being able to produce sufficient milk was experienced as shameful, one of the twin mothers narrated switching to bottle feeds with guilt:

I don’t take enough food due to fever for the past two days so I don’t get good milk. Then I took the babies to private clinic, there the Doctor said, the powder milk is not good for the baby’s health but now you are sick, so for time being you can give powder milk. (lowered head). (Participant 1)

### Theme 2: Kangaroo Mother Care (KMC) – seeing benefits but struggling to practice

Even though the skin-to-skin practice of KMC was new and unfamiliar to the families, they expressed positive attitudes towards KMC. Most families found the environment within the healthcare facility were encouraging especially the visual displays and advice by the health team:

The sister in the ICU helps me… she removes the tubes and places the baby on my chest. There I have been provided with the chair. The doctor has advised KMC for maximum 18 h, minimum 12 h; then only the baby will gain weight. (Participant 3)

The mothers also frequently highlighted the importance of having adequate infrastructure facilities to support KMC, like KMC beds, opportunities for privacy and the inclusion of fathers in the care. Some expressed desire for instructional videos, to learn KMC:

They advise only by talking. If more video shown regarding baby care, it will be good for my family members to see and practice. (Participant 8)

Some fathers also raised concerns regarding the restrictions on men’s access into the NICU and lack of training. This limitation hindered their ability to practice.

Father: If I had been trained, it will be easier for me to hold now. It needs special skill to handle newborn baby. (Participant 5)

Overall, mothers and caregivers articulated several advantages that babies would gain from adoption of KMC. Many of the participants had given birth prematurely, and they expressed the experience of providing KMC is similar to provide womb-like environment for their infants emphasizing the nurturing qualities of the practice.

This method will help the low birth weight baby to grow normal like mother’s womb. (Participant 5)

However, although the mothers and their families had received extensive training and counselling on KMC, many participants did not continue to practice KMC at home.

(Mother smiled shyly): No, we did not practice such thing at my home. The doctor said to do KMC, but we never did. (Participant 4).

The continuum of care from hospital to home largely depends on the support during the discharge process. Some felt that they received counselling support, on the contrary few felt that they received no support.

At the time of discharge, they said I should do KMC…to give breast milk and to take the baby every week to the hospital. (Participant 6).

After discharge families mentioned about minimal support in the community to practice KMC.

The Balwadi (ICDs- Integrated Child Development Scheme) - they give the nutritional mix, the village health nurse - involved with a vaccination. But we don’t get information about the KMC from anybody. (Participant 2)

In addition, many mothers, especially those who had given birth to twins, encountered difficulties in practicing KMC due to post-delivery pain.

When I practice KMC I had headache. Pain over the head and I had especially pain over the neck, it was radiating, and it was pulling my neck. (Participant 1).

Some mothers of LBW infants were also worried practicing KMC might cause harm or discomfort to their babies:

I was scared to carry the baby; I was afraid it was a small baby to keep it close to the chest; I was worried that breathing may stop. (Participant 8)

Balancing household duties with KMC also proved to be a daunting task. Many families found it hard to simultaneously engage in KMC while managing their household responsibilities:

Morning I am not able to do. I have work in the morning. I have to do all the household jobs. (Participant 6)

Finally, the region in Southern India where this study was carried out is characterized by a hot climatic condition, particularly during summer. Families residing in the region expressed various challenges related to the sweltering heat they encountered.

It is hot. We have only a roof- tile roof. It radiates heat inside and it’s very difficult to keep the baby on the chest when it’s very hot. And my baby also not keeping quiet. (Participant 2)

### Theme 3: Family boon or bane - family as a crucial context for community KMC practice

The mothers identified themselves as a part of large family network and the collective responsibility of shared household activities were expressed by them.

The grandmothers played a significant role for the practice of KMC, they served as important caregivers and decisionmakers. Open communication, shared understanding, and a positive relationship between the mother and grandmother were reported to foster a collaborative approach towards KMC:

My mother assisted in the hospital to hold; my mother had always cared for the baby! (Grandma approves and smiles). (Participant 2)

Some grandmothers also took on a leadership role after staying for more than a fortnight in NICU, teaching KMC to new mothers and grandmothers:

I used to talk to other mothers and grandmothers and I teach them the position of KMC, whatever I learnt from doctors and sisters, I will tell others. (Participant 7)

Next to the grandmothers, it was the fathers who took the most active role for KMC in NICU. During the hospital stay where the mothers were convalescing in the post operative ward or intensive care unit, the fathers took active part in doing KMC in NICU:

Father: “I go in the morning between 11 am to 5 pm, give kangaroo care.” (Participant 3).

The hospital had the practice of allowing the men for particular periods of the day and some fathers expressed a wish for more KMC hours in the hospital. Some fathers were emotional to have the baby on the chest and expressed the feelings for KMC:

She had the opportunity to carry the baby in womb. But by KMC I too had the chance. (The father felt emotional his eyes were tearing. He brought the hands to the chest as a gesture of gratefulness). (Participant1)

The mothers also narrated the experience of the other family members in NICU supporting KMC in the hospital:

Two of my sisters came to hospital to do KMC, my elder sister and younger one. Five of our family members came to the hospital and they all did KMC. (Participant 1)

Though the families were supporting in skin-to skin contact and breastfeeding in the hospital, after discharge the dynamics of many families changed. Traditional gender roles with men being breadwinners and the women as babysitters was reinforced by fathers in the community.:

Father: (Looking at the mother, he looked angry): “This is her responsibility to take care of the baby by herself. It is she at home always. If she does not take care as per doctor’s advice, what to do?” (Participant 4).

The traditional gender roles also left a few mothers conflicted in allowing the fathers to handle the babies. Sometimes mothers themselves restricted KMC practice by fathers.

Mother: “I would not allow him to keep the baby on him because he is not gentle with the baby, he does not handle the baby carefully. I am afraid that he cannot do that” (Participant 4).

The mothers also mentioned physical and practical barriers to KMC due to the tradition of mothers moving to their parents’ home after the first childbirth. This meant fathers were visitors when seeing the mother and child, and living standards could be lower than what the father was used to, sometimes constituting a barrier to KMC:

He visits here on Saturday and Sunday only, -- He said,’how long I keep the baby like this — he walks away. (Participant 7)

Burdened by the household chores, many mothers experienced difficulties to practice KMC.

Some of the participants thus felt that they did not get the adequate support for KMC from family members at home:

I alone practice it. I don’t ask my mother. (Participant 5)

But in some families though there was support, the mothers resented the baby being handled by others. The mothers got the idea of cleanliness during the stay in NICU where there is restricted access to visitors. Mothers felt the relatives as a source of infection and emphasized that they should not touch the baby. This aseptic idea was a barrier for KMC care at home, forcing the mothers to manage the burden of household chore and child care alone.

Mother: “Sister, uncle, mother, father, and husband came to the hospital. Only myself and husband should take care. No one should touch the baby.” (Participant 6)

However, some fathers and family members took extra efforts to assist the mother in childcare, finding ways to overcome challenges, like space constraints:

There is no space constraint. If the family is willing all can give Kangaroo Mother Care. (Participant 2)

Some families managed to balance between the outside work, household chores, and the responsibility child care. The enthusiasm of the father and other family members to support KMC at home was an enabler for long hours of KMC practice.

My father keeps the baby in KMC --whoever it may be, all of us in the family cuddle the baby in bed or use KMC in a chair. On average, in a day, 10–12 h we did KMC. (Participant 3)

## Discussion

*When the family support is good*,* continuity of care in the community is better*

The findings from this study provide insight into the family’s experience when they shift care of the newborn from the hospital to the home environment. As shown in the results section, families often acted both as a source of support and as well as conflict to practice KMC at home. A systematic review by Siedman showed that family support, was the top-ranked enabler for continuation of KMC practice in LMICs [28]. In line with this, our results showed that some mothers who continued KMC received extensive support from their families, others continued despite competing household responsibilities. Also, our results nuance previous research on family support and KMC. Some family members were supportive and facilitating of KMC in the hospital, but not necessarily in the context of home, where, as per sociocultural norms, many expected caregiving responsibilities to be handled solely by the mother. Support for KMC after discharge therefore varied considerably.

Similar findings of mothers not feeling supported in continuing KMC by families or communities have also been reported in previous research. A systematic review of KMC highlighted time constraints as a significant barrier [12], a concern echoed by several participants in this study. However, our findings reveal a more nuanced perspective. While some families allocated more time to household responsibilities at the expense of KMC, others devised innovative strategies through collaborative planning. One family, for instance, exemplified this adaptability and devised a structured plan as per work schedule and commuting time; all family members took turns sharing household work and KMC.

Now the question arises: Why do some families brave the odds to continue to practice, while the rest reduce or stop the support? Results from our study reveal that fathers and grandmothers were exposed to KMC in the NICU and played a pivotal role in decision-making, implementing or hindering KMC irrespective of social status. However, in the literature, studies have documented that lack of opportunity to practice is a barrier for fathers to support at home. Fathers in a qualitative study in Australia were unaware that fathers could practice KMC. After they underwent an experiential learning process in the NICU, they supported the process [29]. Similar findings state the importance of Kangaroo Care implementation as a structured education of the fathers [30].

The results from our study showed that some fathers who had experiential learning in the NICU felt emotionally satisfied to be elevated to a maternal role. Similar to our findings, studies from the UK, Sweden, and Denmark have reflected and re-examined the role of father rather than a bystander. When father kangaroo care was instigated, fathers felt empowered to take on equal parenting roles [31–37]. On the contrary, Helth and Jarden [36] found that fathers in a Danish study did not perceive themselves as important as mothers in providing KMC. These disparities are often associated with the different cultural backgrounds. In settings like Zimbabwe, fathers voiced unease about performing KMC because of societal norms that childcare should be the role of the mother [12].

The findings from our study both align with and also contradict the results from existing literature but point to a different direction. Though our study demonstrated father’s assistance in some families, we also found instances where the mother was prejudiced about the father’s ability to handle the small vulnerable newborn; mother herself restricted the father’s participation. This form of “maternal gatekeeping” has been reported in the literature which limits paternal care giving oppurtunities due to mother’s perception and sociocultural norms [38, 39].

One ponders about experience: the counselling sessions in a busy governmental hospital—are they adequate to convince the families, or do they need a more structured program? Literature is abundant with various structural educational programs for members of families, including males. A systematic review by Smith [40] states that supervised practice and educating the family by demonstrations can enhance the confidence of family members to do KMC. Studies from Africa have shown that social support can enhance the uptake and duration of KMC. Many supportive programs in Malawi have utilized the services of respected grandparents to promote KMC. 4000 grandparents were trained in the grandparent’s program, where they provided individual and group counselling in their village to share the messages of positive child rearing [40]. We also saw the resources of grandparents in the current study. One grandmother of one of the participants who stayed for a long time in the hospital took the leadership role and trained recently delivered mothers and became a strong advocate.

There is bias in the literature too, where kangaroo care is often reported as Kangaroo Mother Care, emphasizing the mother’s role. However, research by Blomquist and colleagues [41] shows that when fathers were given the opportunity to practice, they were empowered to provide parental support. This raises an important question: Does Kangaroo Care, when practiced by fathers, yield the same benefits as when practiced by mothers? Studies have found no significant differences in infants’ physiological parameters, such as heart rate, temperature, and oxygen saturation, when fathers provide KMC compared to mothers [42, 43]. This evidence suggests that paternal KMC offers comparable psychological and physiological benefits to maternal KMC, reinforcing the idea that the benefits of skin-to-skin contact and emotional bonding are not gender-dependent. One of the fathers in this study lamented that he had to vacate his place in the Father Kangaroo ward to accommodate mothers due to overcrowding. Another father said that if he was given the opportunity to learn the technique of KMC in the hospital, he would have supported KMC at home. Taken together, the evidence suggests re-evaluating the protocols of neonatal care units, creating training programs, and tailoring interventions and adaptations in the infrastructure to support paternal and other family members inclusion in the neonatal care units to ensure continuity of care at home.

Further, this evidence highlights the need for longitudinal studies to assess family dynamics, paternal mental health, secondary benefits of reduced burden on mothers in high-stress environments, nuclear families, and dual-career families. Policy implications from this study are that the policymakers should consider integrating the findings into public health initiatives, advocating for paternal inclusion in neonatal care practices. Targeted educational campaigns could address societal biases and emphasize the universal benefits of KMC. When families struggle to sustain KMC, they strive to continue breastfeeding but stop skin-to-skin contact.

A palpable tension arises when families feel unable to provide optimal care, reflecting the complex interplay between social and structural factors. Mothers facing breastfeeding challenges adopt various strategies. In low-income settings, alternatives like water or formula feeds are common, and exclusive breastfeeding (EBF) is often perceived as a “western practice” [44, 45]. Conversely, in high-income countries, concerns about milk production and infant weight gain frequently shape feeding decisions. Despite extensive public health advocacy, global EBF rates have remained static at approximately 44 to 48% [46, 47]. Existing literature has documented a recurring pattern of high intentions to breastfeed followed by a shift to formula feeds due to difficulties [48]. A study in Australia has introduced the concept of “breastfeeding grief” as a potential mental health issue for women resorting to formula feeding [49]. One mother in our study, used formula as a temporary measure during her illness, and she felt uneasy discussing her inability to breastfeed her infant.

Though mothers adapted strategies to overcome the difficulties, novel divergent behaviour emerged from this study. While breastfeeding persists despite difficulties, skin-to-skin contact declines or ceases. Much of the existing literature presents breastfeeding and skin-to-skin contact as a unified KMC intervention. However, this study reveals that families adopt distinct approaches to each component, raising critical questions about the underlying drivers of breastfeeding persistence and skin-to-skin contact (SSC). Breastfeeding persistence often stems from immediate cues such as a crying, baby signalling hunger, which makes its necessity more apparent. Conversely, the benefits of SSC are less immediately tangible, as the baby does not actively demand this form of contact. This raises important questions about how families perceive and prioritize SSC compared to breastfeeding, influenced by cultural norms, healthcare education, and support mechanisms.

Breastfeeding is an integral part of the culture and grandmother is a culturally accepted key player who transmits the traditional knowledge across generations. Several studies highlight grandmothers as key influencers in initiating and prolonging breastfeeding [50, 51]. A systematic review by Negin identified that grandmothers’ positive attitudes toward breastfeeding increased initiation rates by 12%, while negative perceptions reduced breastfeeding likelihood by up to 70%. When grandmothers supported breastfeeding, EBF rates increased by 1.6 to 12.4 times, minimizing formula introduction [52]. However, in China, highly educated grandmothers were associated with lower EBF rates, possibly influenced by aggressive formula marketing rather than education itself [53].

Interestingly, in the current study, the integration of traditional knowledge around galactagogues—such as specific foods believed to enhance milk secretion—was prevalent among participants. This phenomenon has been observed in other cultural settings, suggesting a ubiquitous reliance on food and nutrition as essential components of breastfeeding practices among mothers worldwide [54, 55].

Grandmothers in this study actively supported breastfeeding and their role extended to the NICU, where they engaged in KMC practices. While breastfeeding and nutritional supplementation knowledge were culturally embedded, skin-to-skin contact was a novel skill learnt by grandmothers in the NICU.

However, practical challenges have been observed. In a recent narrative review, it is remarked that intermittent KMC, a structured form of SSC, might result in less frequent breastfeeding, potentially impacting infants’ nutritional intake. As infants grow, resistance to the kangaroo position may require adjustments, such as increasing breastfeeding frequency and involving family members in providing SSC to support mothers [56].

An important question arises on how far the grandmothers have imbibed the importance and the art of practising skin to skin contact and transmitted the new knowledge to family members at home? As mentioned earlier, in our study it was feasible to train grandmothers. One in particular took the initiative to transfer her knowledge on KMC to new mothers. However, the same engagement was not achieved among other grandmothers. For further implementation of KMC, there is a need to explore strategies for better involvement of the grandmothers and other family members.

The lack of attention to grandmothers in global health fits into a larger reality of the neglect of elders in the family by the global health community. There is a gap in global health practice and health promotion involving the older generations [56, 57]. Engaging grandmothers can create cascading benefits not only to the child but to the entire families’ health outcome across generations. There are very few public health interventions that can claim such cross-generational impact.

*“Nurturing Confidence: Need to improve frequency of Postnatal visits “*

Postnatal interventions at the community level could play a significant role in facilitating the effective implementation of KMC after discharge. Hadush et al. demonstrated that Community Health Workers (CHWs) in Ethiopia effectively utilized antenatal clinics and postnatal follow-up visits to introduce KMC, supported by community acceptance. They also emphasized that CHWs played a crucial role in KMC promotion (56). Further, a systematic review of 103 publications ranked CHW support as the fourth highest enabler of KMC implementation. In South India, an implementation study on KMC acceleration highlighted the role of CHWs in facilitating family involvement and they used a special link card to support post-discharge communication and continuity of care [56].

Mothers in this study mentioned insufficient postnatal follow-up from CHW on KMC. While they received a nutritional kit, including iron tonic and other supplements, during the antenatal period, these benefits were not extended post-discharge. The findings also pointed towards the gaps in CHW counselling. Participants in this study received counselling and visits during the antenatal period. In these antenatal visits, breastfeeding was a common topic, but many mothers first learned about skin-to-skin care only after delivering their preterm infants. A noticeable disparity between antenatal and postnatal follow-up frequencies emerged, with participants emphasizing the need for structured and regular postnatal visits to integrate breastfeeding and skin-to-skin contact effectively. Considering the vulnerable transition from hospital to home, mothers emphasized the urgent need for continued support from CHWs post discharge.

A study in Ethiopia highlighted that many caregivers have limited knowledge about KMC, which affects their adoption rates; thus, targeted interventions during antenatal and postnatal periods are critical for overcoming these knowledge gaps. According to that study, mothers who received counselling and follow-up visits from healthcare providers demonstrated higher adherence to KMC practices compared to those who did not receive such support [17].

Taken together, there is a need for systematic policies, structured education, empowering CHW and family members creating a holistic support system that enables mothers to sustain KMC.

### Strengths and limitations

This study’s strength lies in its qualitative approach, which captures the nuanced experiences of families practicing KMC. The inclusion of diverse family dynamics and cultural contexts, covering rural, urban, and tribal populations and participants with different socioeconomic status, enhances the trustworthiness of the findings. By observing the transition from hospital to community and including not only mothers, but also fathers and grandmothers, the study provides a comprehensive view of family and healthcare workers’ involvement. While the findings are particularly relevant to LMICs, they might offer transferable insights for diverse sociocultural settings. The specific strategies may differ, but the core principle of inclusive family support remains universally applicable.

Limitations of our study include potential response bias and the contextual specificity of the study. Tamil Nadu, as an advanced state in terms of healthcare in India, limits the generalizability of the findings. Additionally, the primary author’s dual role as both a caregiver in the hospital and a researcher in the field may have influenced the interactions and responses of the families. Further research is needed to explore these findings in varied cultural and socioeconomic settings to solidify their applicability.

### Recommendations for policy and practice

The challenges and enablers identified can inform policies and programs aimed at promoting family-inclusive KMC practices worldwide.

1. Structured Family Education: Develop programs to actively involve fathers and grandmothers, addressing knowledge gaps and empowering families to sustain KMC.
2. Gender-Inclusive Practice and Terminology: Reframe KMC as “Kangaroo Family Care” to challenge traditional norms and promote inclusivity.
3. Enhancing Community Health Worker (CHW) Roles: Train CHWs to counsel families on skin-to-skin contact during antenatal visits and provide post-discharge support to ensure sustained KMC practices.
4. Promoting Cross-Generational Impact: Engage grandmothers as KMC advocates to leverage their influence for cascading benefits across generations.

Health systems worldwide can benefit from incorporating structured educational programs that encourage both maternal and paternal participation in KMC, thus maximizing the potential benefits for infants and families.

## Conclusion

This study contributes to the expanding body of research advocating for inclusive KMC practices that involve all family members, not exclusively mothers. The research highlighted the need to challenge maternal-centric biases prevalent in KMC practices and emphasizes comprehensive family engagement strategies. Promoting gender-inclusive practices, such as rebranding KMC as “Kangaroo Family Care,” is essential, particularly in settings where traditional gender roles may limit paternal engagement. By incorporating structured programs that actively involve fathers and other family members, the overall effectiveness and sustainability of KMC practices could be significantly enhanced. The findings emphasize the critical importance of family support and healthcare workers in sustaining KMC post-discharge. While families demonstrate strong commitment to breastfeeding, maintaining consistent skin-to-skin contact tends to decline over time, reflecting systemic, cultural, and resource-related barriers. This inconsistency stresses the urgent need for structured family education and a shift toward collective family responsibility in newborn care interventions.

Our study also delves into the variability of family engagement during KMC and raises important questions about the adequacy of counselling sessions in busy governmental hospitals. This prompts a critical examination of whether structured educational programs are necessary to effectively engage families in KMC practices. Strengthening community involvement, addressing infrastructural limitations, and enhancing healthcare provider training are vital steps to bridge the gap between hospital and home-based KMC. This holistic approach will foster sustained KMC practices, leading to improved health outcomes for low-birth-weight infants and a more inclusive model of care.

## Supplementary Information

Below is the link to the electronic supplementary material.

Supplementary Material 1

## Data Availability

The dataset used and analysed during the current study is available from the corresponding author on reasonable request.
