# Supplementary material for: Continuation of Kangaroo Mother Care when transitioning from facility to community: maternal and familial perspectives from South India
Source: BMC Health Serv Res. 2025 Nov 5;25:1444. doi: 10.1186/s12913-025-13615-7 (PMC12587497; doi:10.1186/s12913-025-13615-7)
Supplement: Supplementary file 1 — Supplementary Material 1 [file 12913_2025_13615_MOESM1_ESM.docx]

**Low Birth Weight: Continuum of Care in a Community in South India**

**In-depth Interview guide for mothers and other caregivers**

**[Adapted from Lydon et al. (2018)]**

| **Steps for conducting In- depth interview with Mother**  **Step 1:** Complete Screening Questions (I-a to c) in the tool before starting the  interview to confirm eligibility  **Step 2:** At the beginning of the interview, introduce yourself, explain your role in  the study, and thank the mother for her valuable time and willingness to  Share her experience about Kangaroo mother care.  **Step 3:** Read Information and Consent Script to the participant, highlighting the  purpose of the study, confidentiality of responses, voluntary participation  and right to withdraw at any time.  **Step 4:** Complete demographic questionnaire  **Step 5:** Ask for the participants permission to audio-record the interview  **Step 6:** If recording is acceptable, switch on the audio recorder and stat the in-  depth interview using interview guide  **Step 7:** After completion of interview, thank the mother warmly and record end  time. |
| --- |

**I) In-depth Interview details**

| 1. Mother ID : | 1. Interview Date : |
| --- | --- |
| 1. Interviewer Name : | 1. Tape recording Number : |
| 1. Interview start time: | 1. Interview end time : |

**II) Demographic information**

| PICMI ID : |  | Mobile No: |
| --- | --- | --- |
| Mother Name : |  | Father Name: |
| Mother’s age : |  | Father Age : |
| Mother Education : |  | Father Education : |
| Mother’s occupation(if employed): |  | Father Occupation : |
| Mother’s Income (Monthly) : |  | Father Income (monthly) : |
| Total No. of children delivered : |  |  |
| Type of delivery (current) : |  | Household Type: Thatched/ Tiled/ Pucca/ others………….) |
| Source of Infant Information: ……………… |  | Religion : Hindu/ Muslim/ Christian |
| Name of the baby: |  | Total No. of Living children |
| Age of baby : |  | Place of Delivery: Private / Govt. PHC /GTH/GH/GMCH/Others…………….) |
| Sex of baby : |  | Date of discharge: |
| Birth weight : |  | No. of days stay at SNCU |
| Gestational age : |  | Duration of KMC in SNCU (in days): |
| Street : |  | Place of residence |
| Village : |  | HSC : |
| Block : |  | District :Dindigul/Theni/Kancheepuram |

| **Introduction and Rapport**  Can you tell me about your pregnancy and delivery experience in the hospital?  **Probes:**   1. Ante natal care 2. Tell me about your baby’s birth- When? Where? and transport 3. How was your health after delivery? 4. How was your baby’s condition in the first week at home? |
| --- |

1. What do you understand by the term low birth weight baby?
2. What type of care should be given to babies who are born before the expected date of

delivery?

Probe

- 1. From where did you learn to care about newborn babies?
  2. Who informed you on newborn care?
  3. What did they say?

1. What does your culture say about babies being born before expected time of delivery?
2. Who are the other family members involved in child rearing?
3. Did you take advice from anybody or discussed with anybody about this baby? Probe
   1. Who did you discuss with
   2. What did you discuss about?
   3. What did the person tell you or advise you?
4. Can you explain about your experience in the SNCU ward about KMC and breast feeding practices

Probe:

- 1. Who are all taught you about KMC?
  2. Sling bag use
  3. Who are all practiced KMC from your family during hospital stay?
  4. Explain about the challenges faced by you in KMC and breastfeeding

1. Do you know about KMC? If yes, what is it? How helpful do you suppose it is?

Probe

- 1. When did you find out about KMC? During antenatal or postnatal period?
  2. From where did you learn about KMC? Who informed you about this?

1. What do you know about the procedure of KMC?
2. What do you think about KMC for pre-term babies?

Probe: Do you think KMC is beneficial? Why or why not?

1. What is your experience regarding the counselling services? Did you receive any pre-

discharge counselling about community continuation of KMC practices?

Probe:

- 1. What did they talk about in the counselling session?
  2. Did you understand the message? Was it helpful?
  3. Was it appropriately delivered? Was it interactive?
  4. Please explain and messages or advice?
  5. What were barriers and challenges faced whiling receiving the counselling services

1. Were there any barriers to practicing KMC at health care centre? If yes, what were they

and how did overcome it? Apart from skin-to-skin care, how else do you keep the baby

warm?

- 1. Do you have enough space to practice KMC?
  2. Do you have privacy?
  3. Do you have a comfortable separate chair bed etc.?

1. How did you first start breastfeeding your baby after delivery?

Probe

a. Was it immediate or delayed?

b. What were the reasons?

1. Did anyone guide or support you?
2. Can you tell me a little about your baby and your experience after discharge from the hospital?

Probe

- 1. How was your health after delivery?
  2. How was your baby’s condition in the first week at home?
  3. How did you care for the baby at home after discharge?

1. Do you practice KMC at home? If yes, how long and how many times do you provide

KMC?

1. What do you think are the cultural barriers to practicing KMC at your home after discharge? Do you think this against your cultural beliefs?
2. Are there any personal discomforts or psychological barriers in practicing KMC at home?
3. Who practices KMC at home in case of your inability?
4. What does your community say when other family members practice KMC?
5. Do you get support from your husband and other family members for practicing KMC?
6. Who is the deciding authority or the head of your family?

Probe: Do they intervene or support on the practice of KMC?

1. Is your house comfortable for providing KMC?

Probe

- 1. Do you have enough space in the home to practice KMC?
  2. Do you have privacy at home? E.g. a separate or personal room?
  3. Do you have a comfortable place at home to provide KMC (like separate mat, bed or chair)?

1. Does your household work or other work affect your practice of KMC?
2. Do you realize any benefits from practicing KMC?

Probe

- 1. Has your lactation improved after KMC?
  2. Has your relationship with your baby improved after providing KMC?
  3. Does KMC help to reduce your anxiety on the baby’s health?
  4. Do you think your baby’s health has improved because of KMC?

1. Do financial barriers influence the practice of KMC at home?
2. Are there any leaders in your community who advocate or are against KMC?

Probe: Does their opinion influence you and your family’s opinion on KMC practice in community?

1. What can facilitate continuation of KMC at home after being discharged from the

health facility?

1. Who can support KMC in the communities and how?

Probe: Role of family members, community leaders, community health care service providers

1. What is the mechanism of post discharge community follow up of preterm babies

after receiving facility based KMC services?

Probe

- 1. Did anybody visit your household to follow up on your preterm baby?
  2. Who visited? When was the last time they visited? What did they do during his visit?

1. Did you receive any help from other mothers regarding KMC at hospital or

community?

1. What do you think of a KMC peer mentoring? Would you want to be supported by another woman who has practiced KMC? Why? Why not?
2. Do you think KMC is a feasible practice in the community?
3. What challenges did you face in breastfeeding?

Probe

Sickness of baby or mother, less milk secretion /barrow from others or milk bank,

Latching problems, Nipple issues, Advice and diet support for milk secretion

1. Can you describe your breastfeeding routine?

Probe

Frequency, Day/night feeding, Exclusive breastfeeding vs other feeds

1. Were there times you felt like stopping or reducing breastfeeding?
2. What do you usually do in your community to increase breast milk secretion?

Probe

a. Traditional delivery medicines

b. lactogenic foods

c. other beliefs and food practice

**---------------------------------**
